# Child Life under Queen Victoria

**Published:** 1897-04-03

**Authors:** 


### CHILD LIFE UNDER QUEEN VICTORIA.

### VIII.—RAGGED AND INDUSTRIAL SCHOOLS.

ONE John Falk, a German, is said to have been the originator of ragged schools. At the close of the Napoleonic wars Falk was distressed at the number of soldiers' orphans who, if friendless, often lapsed into crime. He gathered as many as he could together, taught them useful trades, and altogether humanised them. As he says, "where chains and stocks, the lash and prison, were powerless, love comes off victorious." About the same time Thomas Cranfield, who had himself been a soldier, was doing similar work in England. These efforts were, however, small and local. The ragged school movement took its first great start in Scotland, largely through the efforts of Dr. Guthrie, a well-known clergyman. In England the work is largely associated, as so many good works have been, with the name of Lord Shaftesbury. He was not the founder of ragged schools, but from the day in February, 1843, when he saw an advertisement in the *Times* inviting ladies and gentlemen to help in the ragged school at Saffron Hill, he was in the forefront of the movement.

The particular school which drew Lord Shaftesbury's attention was situated in what was called in the slang of the neighbourhood "Jack Ketch's Warren," because so many of the inhabitants ended their career on the gallows. Concerning the district a missionary writer* says: "The disturbances here were of so desperate a character, that from forty to fifty constables would be marched down with cutlasses, it being frequently impossible for officers to act in fewer numbers or unarmed." The first beginnings of a school in such a neighbourhood could not be encouraging. Charles Dickens, who visited the school, thus describes it: "It was held in a low-roofed den, in a sickening atmosphere, in the midst of taint, and dirt, and pestilence; with all the deadly sins let loose, howling and shrieking at the doors. Zeal did not supply the place of method and training; the teachers knew little of their office; the pupils with an evil sharpness found them out, got the better of them, derided them, made blasphemous answers to Scriptural questions, sang, fought, danced, robbed each other — seemed possessed by legions of devils." So much for the conduct of the pupils. Lord Shaftesbury himself bears testimony to their filthiness. "Frequent are the occasions," he says, "on which the female teachers have returned to their homes covered with the vermin of their tattered pupils." Great must have been the devotion that kept teachers at work in such an environment. They were rewarded, however. To quote from Dickens again, he states that on a subsequent visit he found this same school in Saffron Hill "quiet and orderly, full, lighted with gas, well whitewashed, numerously attended, and thoroughly established."

The first intention of ragged schools was to make up in some degree for the lack of opportunities for education among the less fortunate classes of the community, and also to give the pupils religious instruction —to teach them "just the meaning of their sorrow," as Mrs. Browning put it. State education has relieved the ragged schools from the necessity of teaching the "three R's," but they still hold their place in the ranks of evening schools, where a wider range of subjects is taught than in day-schools, with less exactness, perhaps, but in a more attractive manner.

Moreover, they have been the parents of philanthropic effort in many directions. One thing, of no small national importance, may claim to have sprung directly from the founders of ragged schools coming in contact with homeless and destitute children. It was in 1866 that Lord Shaftesbury persuaded the Government to grant the *Chichester*, a disused fifty gun frigate, for a training ship for boys who were willing to be trained for the royal or the merchant navy. Training ships are now an integral part of our national economy, and are the means of training up to an honourable life many who would otherwise have had no outlet for their energies but crime. Indeed, homes for destitute children, where they are trained in one or another useful occupation, form seed-plots whence our colonies are largely peopled.

Ragged schools are in themselves voluntary institutions, but we may associate with them industrial schools, which, though they derive some part of their income from private benevolence, are more directly under Government control, and receive grants from the Treasury as well as subsidies from local rates. Industrial schools must not be confounded with reformatories. The children at industrial schools are not as a rule criminals, though they are often the children of criminals. If a child is found homeless or destitute, or trying to live by begging, anyone who so discovers him may take him before a magistrate, who can order him to be sent to an industrial school. Many of the children are orphans, but many have one or both parents in prison. Moreover, any child who is found in the company of thieves or disreputable persons may be sent there. Refractory children are occasionally sent either from their homes or from the workhouse to industrial schools, and children under twelve years of age who have been convicted of a criminal offence. Different industrial schools are under the control of different religious bodies, and where it is possible a child is sent to a school under the charge of the denomination to which its parents profess to belong. There are also day industrial schools for those children whom it is undesirable to send to ordinary schools. Confirmed truants also may be sent to industrial schools. If, however, a parent asks to have his child sent to an industrial school, he may be required to contribute to his maintenance and training a sum not exceeding five shillings a week. The time for which the child is committed is left to the discretion of the magistrate who sends him, but he cannot be detained after he is sixteen years old.

Reformatory schools are intended for children of more pronounced criminal instincts. They are the outcome of the efforts of the Philanthropic Society, of which Sir Stafford Northcote (Lord Iddesleigh) was one of the most prominent members. An Act was passed in 1854 "for the better care and reformation of youthful offenders in Great Britain." Other Acts developing, improving, and consolidating the system have since been passed. Each child in a reformatory costs the Government about 6s. This does not cover the whole expense, the remainder, about 1s. 6d., being taken from the local rate. If this money prevents young offenders from becoming permanent members of the criminal class it is certainly well spent.

* R. W. Vanderkiste, quoted in Hodder's "Life of Lord Shaftesbury."

